# Designing novel Sn-Bi, Si-C and Ge-C nanostructures, using simple theoretical chemical similarities

**DOI:** 10.1186/1556-276X-6-362

**Published:** 2011-04-27

**Authors:** Aristides D Zdetsis

**Affiliations:** 1 Department of Physics University of Patras, GR 26500, Patra, Greece

## Abstract

A framework of simple, transparent and powerful concepts is presented which is based on isoelectronic (or isovalent) principles, analogies, regularities and similarities. These analogies could be considered as conceptual extensions of the periodical table of the elements, assuming that two atoms or molecules having the same number of valence electrons would be expected to have similar or homologous properties. In addition, such similar moieties should be able, in principle, to replace each other in more complex structures and nanocomposites. This is only partly true and only occurs under certain conditions which are investigated and reviewed here. When successful, these concepts are very powerful and transparent, leading to a large variety of nanomaterials based on Si and other group 14 elements, similar to well known and well studied analogous materials based on boron and carbon. Such nanomaterias designed *in silico *include, among many others, Si-C, Sn-Bi, Si-C and Ge-C clusters, rings, nanowheels, nanorodes, nanocages and multidecker sandwiches, as well as silicon planar rings and fullerenes similar to the analogous sp^2 ^bonding carbon structures. It is shown that this pedagogically simple and transparent framework can lead to an endless variety of novel and functional nanomaterials with important potential applications in nanotechnology, nanomedicine and nanobiology. Some of the so called predicted structures have been already synthesized, not necessarily with the same rational and motivation. Finally, it is anticipated that such powerful and transparent rules and analogies, in addition to their predictive power, could also lead to far-reaching interpretations and a deeper understanding of already known results and information.

## Background

**Figure 1:**
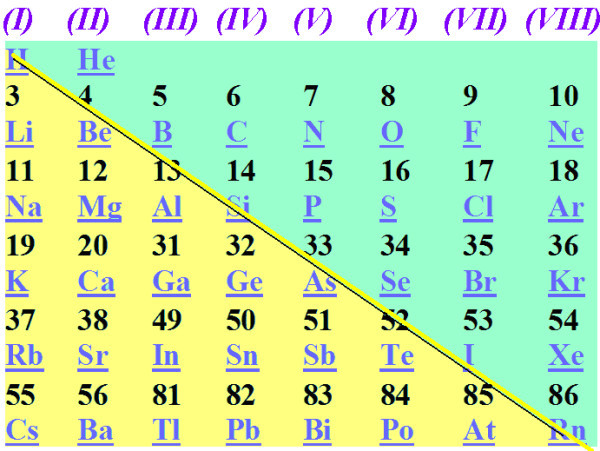
**The periodical table of the main group elements**. The diagonal line roughly separates metals and non-metals.

There are several very powerful, general and fruitful concepts in physics and chemistry which are, at the same time, miraculously simple and highly efficient for the molecular engineering and design of functional and functionalizable nanomaterials and nanosystems. Such transparent and powerful concepts are based on isoelectronic (or isovalent) analogies, regularities and similarities, of which the periodical table of the elements is the best, simplest and most celebrated example. The periodical table constitutes a global regularity. Another much less known global regularity is illustrated in Figure 1 that shows the main part of an older version of the periodical table which includes only the main group elements without the d-block and the f-block elements. If we draw a diagonal in the table in Figure 1 we can see that all (or most) elements below the diagonal are metals and that the elements above the diagonal are non-metals [1]. An elementary explanation is that the elements on the left have only a few valence electrons which they can easily loose, whereas elements on the bottom have several inner shells of electrons which screen up and weaken the nuclear attraction to the valence electrons and which can, therefore, escape easily into the (free) electron sea.

Similarly, there is another global diagonal relationship between elements (composed mainly of the third and second periods) which demands that diagonally adjacent elements (primarily of the second and third periods) have similar properties (in particular those related to size and electronegativity) [2,3]. The size of the atoms decreases when moving across a period of the periodic table and increases when moving down a group. Likewise, the electronegativity increases when moving across a period and the elements become progressively more covalent and more electronegative. On moving down a group, the elements become more ionic and less electronegative. Thus, crossing and descending the periodic table have opposite effects on these properties, cancelling out for diagonal pairs of elements such as B and Si (for instance, boron and silicon are both semiconductors). Similarly, and in addition to the diagonal relationship there is an obvious vertical relation between elements of the same column of the periodical table (groups) which have the same number of valence electrons and similar (more or less) chemical properties. Yet, as we descend down a group, there are systematic and regular differences, described by the well known inert pair effect [2-5] - that is, the increasing stability of oxidation states that are two less than the group valency, for heavier elements of the group. It can be also described as the resistance to losing the s-electron in the valence shell (for the group 13, 14 and 15 elements). This is a relativistic effect which is due to the better penetration of the s-valence electrons to the nucleus compared to p-electrons [2-5].

**Figure 2:**
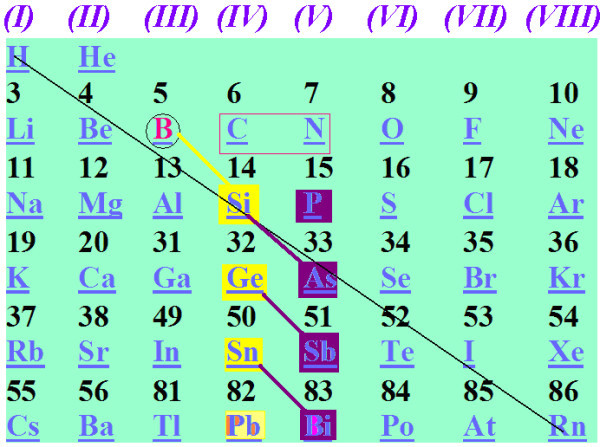
**The region of the periodical table examined here**. Different colours of diagonal lines and element symbols signify different type or level of (co)relation.

In addition to such global regularities, there could be other local analogies which are also based on the number of valence electrons. In general, it would be expected that two atoms or molecules having the same number of valence electrons (isovalent) should have similar properties and should, in principle, be able to replace each other in larger complex structures and nanocomposites. Such a local diagonal relationship between boron and silicon can be invoked by recognizing that, basically, one B-H unit has the same number (four) of valence electrons (is isovalent) with Si. We could then assume that, under certain conditions to be specified later, we could replace a B-H unit in a boron hydride molecule by Si and still have a stable molecule. We could also expect that the reverse could be also true but we will not consider it here. Let us write the above relation as BH→Si (1). It is clear that isovalency, although necessary, is not sufficient for such equivalence or replacement relationships. For instance, we cannot write Si→C in general. In addition, the relationship BH→C does not work either. Experience shows that we could, instead, have BH^1-^→CH (2). This is a horizontal relation involving five valence electrons. The relation (CH)→(SiH) (3) also seems to works as C_20_H_20 _or C_60_H_60 _fulleranes are very similar to Si_20_H_20 _or Si_60_H_60 _fullerens [6-9]. Relation (3) could be also written as (CH_4_)→(SiH_4_) (3') to indicate the equivalence covalent (sp^3 ^bonded structures). Moreover, since Si, Ge, Sn and Pb are in the same column of the periodical table, we could, in principle, write: Si→Ge→Sn→Pb (4), bearing in mind the inert pair effect, although it is clear that Si and Pb are not similar. However, it is not unreasonable to expect that (BH)→Ge, (BH)→Sn, (BH)→Pb (5) would work as a total substitution (which is roughly correct). Relation (5) involves four valence electrons. As in relation (2), which involves five valence electrons, we can write more five-valence electron relations as: BH^1-^→CH→P, BH^1-^→CH→As (6) or BH^1-^→CH→Sb, BH^1-^→CH→Bi (7), involving the group 15 elements. Relations (1) and (2) [10-16] and analogy (3) [6-9] have been successfully tested. The author has also tested CH→Si^1- ^(14) which has lead to a simple rule of thumb for constructing planar aromatic Si structures similar to benzene and others [17-19]. This rule, suggested by Zdetsis, has been also tested and verified by Jin *et al. *[20]. Finally, analogies (4) through (7) have been demonstrated to be valid by the author [21-24]. Thus, in this study, the working area of the periodic table will be as in Figure 2. Even within this limited region of the periodical table, it is difficult to identify similar or homologous molecular systems.

The obvious questions are how could one know beforehand which of the many conceivable similarities (or equivalence relations) are valid (or working) and what is the criterion or criteria?

A natural, but incomplete, answer should certainly depend on chemical (and physical) intuition and plain common sense, although sometimes in science common sense has lead to wrong conclusions. This approach suggests that not only the number of valence electrons but also the type of bonding (hybridization, relative magnitude) should be important for such similarities. For instance, carbon in diamond and crystalline silicon have similar properties because they are both sp^3 ^bonded through strong covalent bonds. In such a case the replacement Si→C is valid but not in the case of graphite (which is sp^2 ^bonded) or benzene (which is aromatically bonded). In this case we can derive the rule CH→Si^1- ^(8), by noting that the Si_6_^6- ^multianion has a planar hexagonal structure similar to benzene (C_6_H_6_) [17,18],from which it follows that Si_6_^6-^→C_6_H_6 _or CH→Si^1- ^(8). Both of these relations are isovalent (involve the same number of valence electrons and can be realized [17,18] by placing lithium counter-ions in suitable positions. This has been known as the Si_6_Li_6_→C_6_H_6 _(9) replacement rule of thumb. Such bonding properties are reflected in the structure, symmetry and occupation of the frontier orbitals through which these simple ideas can be strictly formalized, classified and theoretically implemented. Analogous ideas have been applied for the silicon and carbon fullerenes [6-9] which are similar because they are similarly sp^3 ^bonded (not sp^2 ^as in the real carbon fullerenes). Such a formulation has lead to the *isolobal *principle (analogy) [25]. The isolobal analogy was introduced about 30 years ago by Hoffman [25] in order to allow the correlation of seemingly very different chemical species, such as organic hydrocarbon fragments, with transition metal ligands. Two fragments are isolobal if the number, symmetry properties, approximate energy and shape of the frontier orbitals, as well as the number of electrons in them, are similar (but not identical). Although it seems strange, CH, Co(CO)_3 _or NH_3_, Co(CO)_4 _can be considered as classical examples of pairs of isolobal fragments. Closer examination reveals that all these fragments need the same number of missing electrons in order to reach a stable electronic configuration. For example, CH has five valence electrons and, therefore, it needs three more to reach the stable configuration of eight. Likewise, Co(CO)_3 _which has 15 valence electrons misses the stable configuration of 18 by three electrons. These fragments or building blocks can replace each other in more complex structures and can combine to form ordinary bonds.

The isolobal analogy allows us to relate and compare organic, inorganic and/or organometallic compounds on a uniform basis. Inversely, if we can find molecular fragments or building blocks that can replace each other in complex structures, we can conclude that they are isolobal. Therefore, the best way to examine and validate any (local) chemical similarity, discovered by chemical intuition and/or general concepts (based on isoelectronic or isovalent analogies) is the scheme demanded by the isolobal principle. The replacement BH→Si (1) and most of the analogies (1)-(7) involving (directly or indirectly) boron are special cases of a more general isolobal analogy which the author, scoptically and synoptically, has termed 'the boron connection'. This analogy provides a mechanism to predict new, hopefully stable, molecules and to compare molecular fragments with each other and with familiar species from organic chemistry and also offers clues about reactivity and reaction mechanisms. The boron connection is an attempt to map the structural chemistry of boranes and carboranes [26-31] to the structural chemistry of silicon [32] and silicon-carbon clusters, respectively. Boranes and carboranes constitute a very rich and well-established branch of chemistry with well-known structural and stability rules and powerful concepts [26-30]. Since boranes and borane-based molecular fragments and nano composites - including carboranes, bisboranes and metallaboranes - and metallacarboranes are very well studied [26-31,33-35] and are very well known species with many technological, chemical and biomedical [33,34] applications, their silicon, silicon-carbon (and other group 14, and 15) analogues [35,36] are expected to be very important and very promising for analogous applications.

It should be emphasized that not all of the similarities and results obtained were initially conceived as such but they have later been unified, classified and generalized within the framework and the scope of the present work. Clearly, not all the results presented here are reviews of earlier work. Several totally new results and predictions have been obtained here.

## Results and discussion

### The boron connection for Si and Si-C structures

**Figure 3:**
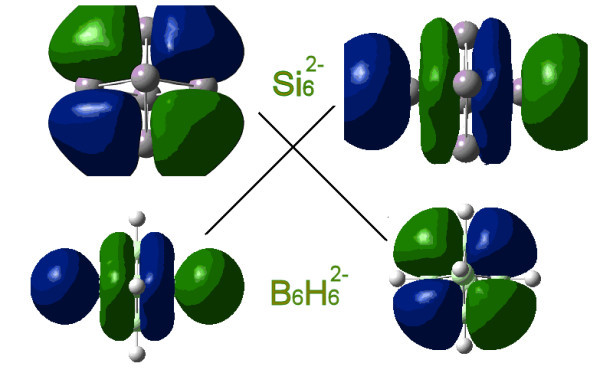
**Schematic illustration of the original boron connection between dianions**. Isolobal analogy of Si_6_^2- ^dianion and the B_6_H_6_^2- ^borane.

**Figure 4:**
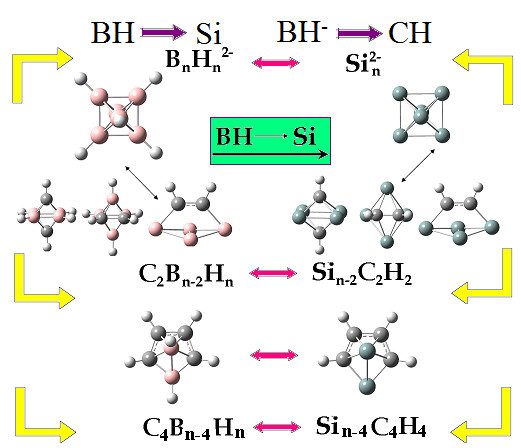
**Schematic illustration of the SiC- extended boron connection**. Isolobal analogy of Si_n_^2-^, (B_n_H_n_) ^2-^, Si_n-2_C_2_H_2_, C_2_B_n-2_H_n_, including the Si_n-4_C_4_H_4 _and C_4_B_n-4_H_n _similarity.

**Figure 5:**
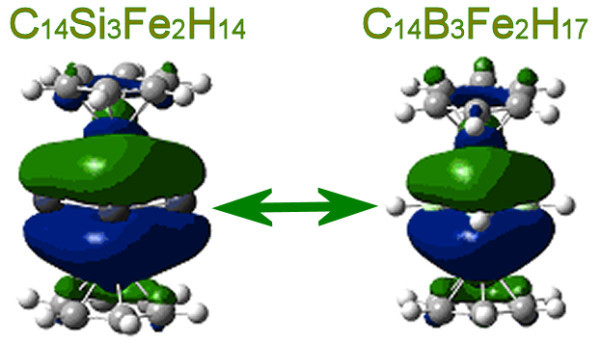
**Illustrating the isolobal analogy of multidecker sandwiches**. Comparison of the HOMO orbitals of Si-C and B-C multidecker sandwiches.

The BH→Si boron connection has originated from the fluxionality and similarity of magic silicon clusters (and, in particular, of the controversial Si_6 _cluster [11-13]) with the corresponding deltahedral boranes. It was shown initially [11,12] that the Si_6_^2- ^dianion and the corresponding isovalent B_6_H_6_^2- ^borane have exactly the same geometrical and electronic structure (including the frontier orbitals) as shown in Figure 3 and they are, therefore, isolobal and homologous. Furthermore, through the BH^1-^→CH (2) or 2BH^1-^→ 2CH substitution, the validity of which is established through the synthesis and stability of the well-known C_2_B_4_H_6 _(and, in general, of C_2_B_n-2_H_n_) carborane, we can assume the isolobal equivalence Si_n-2_C_2_H_2_⇔C_2_B_n-2_H_n _(10) between neutral hydrogenated silicon carbon clusters and deltahedral carboranes [10-15]. The same is true for Si_n-4_C_4_H_4 _clusters and C_4_B_n-4_H_n _carboranes (namely Si_n-4_C_4_H_4_⇔C_4_B_n-4_H_n_), as illustrated schematically in Figure 4. Applying the isolobal analogy (10) to existing organometallic multidecker sandwiches [30-33] we have designed (*in-silico*) analogous organometallic silicon- carbon sandwiches[16] which, as shown in Figure 5, are fully homologous (isolobal) to the carborane prototypes and are, therefore, expected to have similar chemical and technological properties. Apparently similar results should be able to be obtained for germanium-based multidecker sandwiches, since Si and Ge structures are fully homologous and isolobal and the Si→Ge substitution rule is valid almost everywhere. This is equivalent to the BH→Ge substitution, which seems to be more valid compared to the BH→Si substitution, in particular for larger clusters [12,13]. This is related to the fact that the BH→Si (1) is based on the much stronger BH^2-^→Si^2- ^substitution between dianions [10,11] from which the equivalence Si_n-2_C_2_H_2_⇔C_2_B_n-2_H_n _is obtained by the well-tested substitutions Si1^-^→CH and BH^1-^→CH, respectively. It is the BH^2-^→Ge^2- ^relation which is much more valid when compared to the BH^2-^→Si^2^, mainly due to the inert pair effect [14,15]. For exactly the same reason, as we go down the 14th column of the periodical table (see Figure 2), the BH^2-^→Sn^2- ^relation is even more valid than the BH^2-^→Ge^2-^, which is verified experimentally by the recent synthesis of stannaspherene, Sn_12_^2- ^(see Discussion section) [14,15,22].

**Figure 6:**
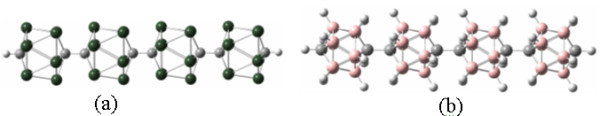
**Comparison of silicon-carbon and boron carbon nanorods**. (a) Si-C and (b) B-C nanorods.

In addition, Hawthorne and collaborators [34,35] have designed and synthesized special carborodes made of chains of icosahedral carboranes interconnected to each other, as shown in Figure 6b, for biomedical applications as drug delivery and radiolabelling agents. The corresponding isostructural and isolobal Si-C nanorods, shown in Figure 6a, are expected to have similar properties and capabilities. The added advantage of such systems, which are based in organosilicon chemistry in analogy to organoboron chemistry, is the lower toxicity and expected higher biocompatibility [35] and biodegradability [36] of silicon. In addition, the possibility for the integration of the diagnostic and therapeutic tools with the current micro- and nano-electronic tools and infrastructure based in silicon technology is very appealing. Such a possibility was first suggested by Zdetsis [22,23]. Finally, it has been shown that each separate unit of the Si-C nanorodes, after the removal of the apical hydrogen atoms, can exist as a separate, independent unit in a form of a nanowheel, similar to the B-C wheel-shaped structures [37,38]. As a test for the predictive power of the boron connection scheme, we have, in parallel to the current investigation, started a search for such Si-C based nanowheels which has been successfully finalized [38]. This is highly suggestive of the endless variety of molecular engineering possibilities of such simple-looking analogies.

### The boron connection for Sn, Bi and Sn-Bi structures

**Figure 7:**
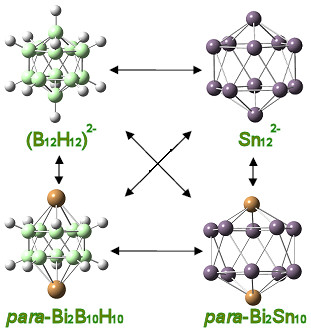
**Schematic illustration of the CH → Bi/BH → Sn analogy**. Correspondence of stannaspherene (Sn_12_)^2-^ with (B_12_H_12_)^2- ^and analogous bismuth functionalized structures.

**Figure 8:**
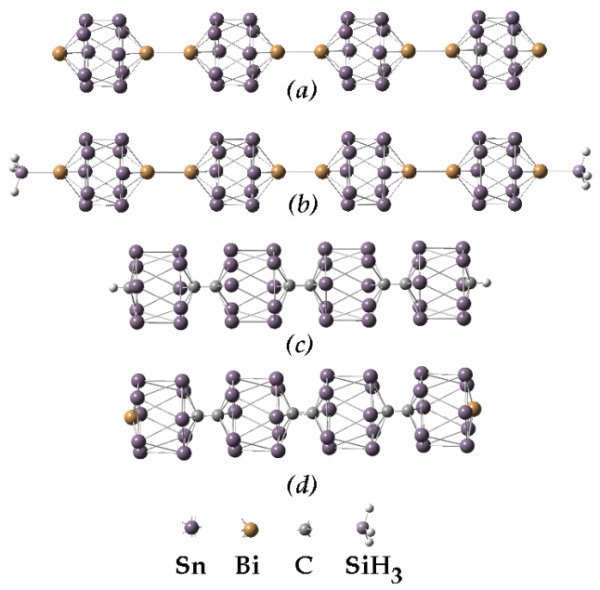
**Comparison of a larger verity of nanorods involving group 15 elements**. Si-C, Sn-Bi, and SN-C-Bi functionalized nanorods.

**Figure 9:**
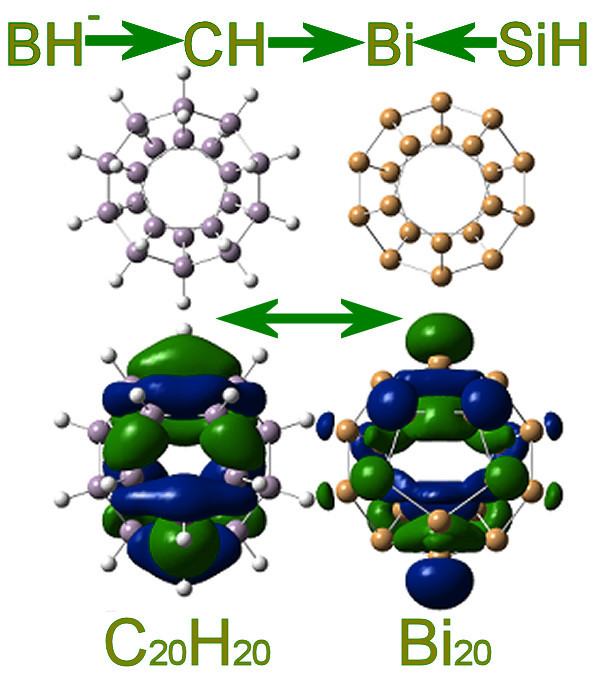
**Illustration of the Bi/C fullerenes analogy**. Comparison and analogy in geometrical and electronic structure of C_20_H_20 _and Bi_20 _fullerenes.

The equivalent isoelectronic relations rules BH→Sn and BH^1-^→Bi. could be also considered in a similar way to the BH→Si and BH^1-^→CH rules: The latter analogy is effective in the synthesis of bisboranes, while the former is based on the equivalence of Si and Sn (belonging to the same group) and the boron connection. Such equivalence, as shown in Figure 7, was verified by the recent synthesis of the Sn_12_^2- ^dianion, the stannaspherene [21,39]. Through the stannaspherene synthesis, the possibility of forming functional nanostructures similar to hydrogenated silicon carbon nanorods and carborods is highly enhanced. The nanorods shown in Figure 8 are very stable (high binding energies) and homologous to each other. In addition, due to the formal equivalence of CH and SiH (in cases of sp^3 ^bonding), we can assume the BH^1-^→CH→SiH replacement and examine the possibility of bifullerenes such as Bi_20_, in a similar manner to the corresponding C_20_H_20 _or Si_20_H_20 _fullerenes. As shown in Figure 9, the two types of fullerens are fully isolobal, which is highly suggestive that such bismuth cages could be eventually synthesized [40]. Moreover, since Si^1- ^ions (or P atoms) are very popular doppands of C_20_H_20 _cages, forming Si^1-^@C_20_H_20 _or P@C_20_H_20 _embedded cages, we could expect that Bi@Bi_20 _cages would also be stable and isolobal to Si^1-^@C_20_H_20_. This is indeed the case [23]. Similar results have been obtained for P_20, P@P20 _or P@Bi_20 _cages, and so on [23]. It is further predicted and anticipated that nanostructures involving other elements or other combinations of group 14 and group 15 elements could, in principle, be realizable within the reservations expressed above (for example between C and Si) and the limitations due to the inert pair effect [10-15].

### (CH)→(SiH) or (CH_4_)→(SiH_4_) substitution: Si fullerenes

**Figure 10:**
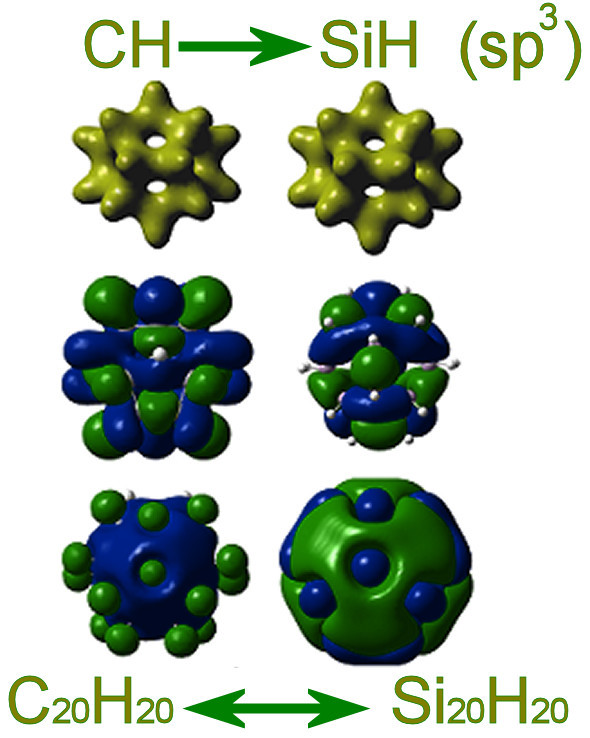
**An illustration of the Si and C fullerenes analogy**. Comparison and analogy in geometrical and electronic structure of C_20_H_20 _and Si_20_H_20 _fullerenes.

The C_20_H_20 _or C_60_H_60 _hydrogenated fullerenes (or fulleranes), unlike the bare C_20 _and C_60 _cages, are sp^3 ^bonded and, therefore, homologous Si_20_H_20 _or Si_60_H_60 _fullerenes should, in principle, be stable and isolobal to C_20_H_20 _or C_60_H_60 _[40] and this is, indeed, valid. Silicon fullerenes on the basis of their binding energies are, in fact, very stable [6-9,40] and isolobal to the corresponding isovalent carbon fulleranes, as is indicated in Figure 10.

### The CH→Si^1- ^or CH→Si^1-^Li^1+ ^rule of thumb: planar aromatic silicon rings

**Figure 11:**
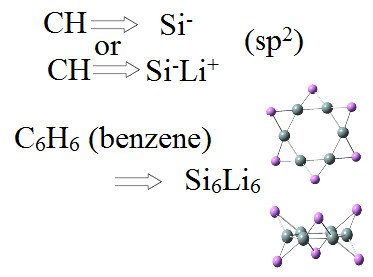
**An illustration of the CH/SiLi analogy for planar aromatic Si-based structures**. Top and bottom views of the planar Si_6_Li_6 _aromatic structure similar to benzene.

**Figure 12:**
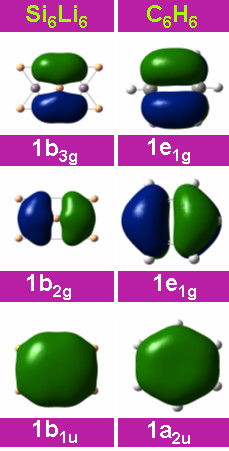
**Comparison of the electronic structure of Si_6_Li_6 _and benzene**. Comparison of the frontier orbitals of Si_6_Li_6 _and Benzene, showing the isolobal analogy between the two.

As explained earlier, for the planar aromatic carbon rings, the corresponding planar Si structures are obtained from the SILI rule of thumb [17-20], as illustrated in Figure 11. The resulting structure in Figure 11, is very stable (not necessarily the global minimum structure), aromatic [17-19] and fully isolobal to benzene as is shown in Figure 12. It is interesting to observe that, in this case, although isolobal, the two rings are not isostructural (they do not have the same structure and symmetry [17,18]. Furthermore, following this rule of thumb suggested by the present author [17,18], Jin and Liu [20] have recently designed larger planar rings (analogous to naphthalene or coronene). Such rings are both stable (high binding energies, real frequencies) and aromatic, which is very promising for a possible future synthesis of such planar Si structures.

### Other possible applications and implications in nano-medicine and biology

In this work we have been mainly concerned with total substitutions of the form BH→Si, Si→C, Si→Sn, BH^1-^→CH, and CH→Bi and others. However, partial substitutions could be as (or even more) useful as total substitutions. Such partial substitutions, which have not so far been investigated or tested (and, thus, constitute new global predictions of the current paper) could be very useful in medical and pharmaceutical applications, such as drug delivery, radioimaging and radiolabelling, among others. The building blocks of such functionalized nanosystems are borane ions, carboranes, metallacarboranes, metallaboranes and other heteroatom derivatives or combinations. For example, mixed BHC and SiC nanorods, obtained by partial (not total) BH→Si substation in the model systems discussed above, should be able to be used for radiolabelling by iodination or tritiation, respectively, as described by Hawthhome and Maderna [33,34]. Such a possibility has recently been successfully explored [41] with very promising preliminary results. In all these analogous material symmetry is very important and should be taken into account, provided there are additional tests to verify that no imaginary frequency modes occur, leading unavoidable to distortions (as in the case of Si_12_^2-^).

Finally, the recent (well after this work was finalized) discovery of *A Bacterium That Can Grow by Using Arsenic Instead of Phosphorus *by Wolfe-Simon *et al. *[42], indicates that such rather simple and 'innocent' isovalent (or isolobal) substations used throughout this work, could have very far-reaching implications in many branches of science and technology.

## Conclusions

It has been illustrated that chemical intuition supported by rather simple and transparent, but very powerful techniques, can form a general scheme or framework leading to molecular engineering and theoretical design of very important and functional nanomaterials and nanostructures based on silicon, carbon and other group14 and group 15 elements. It has been also illustrated that the framework presented here, in addition to the older and newer predictions (some of which have been already tested and/or materialized), could lead to very far-reaching implications and applications in many branches of science and technology, including nanomedicine and molecular biology.

Yet, its implementation is not always unique or straightforward, due to many alternative misleading routes. Chemical (and physical) intuition is always very important. Apparently, the same scheme, based on observed and well-defined properties of one category of materials should be able to predict and design analogous materials, with similar chemical properties for a (seemingly) different category of structures. We have restricted our attention to group 14 (and group 15) elements and structures (based on well-defined properties of group 13 elements and, in particular, boron) due to the high technological importance of silicon and carbon (and the other connected elements and fragments). Even within this limited range of the periodical table (illustrated in Figure 2), the possibilities are endless and the possible routes unlimited. Hopefully, the scheme presented here should be able to guide the search, the theoretical design and the validation (through the isolobal criteria) of the (theoretical) results. Obviously (and unavoidably), experiment and technological synthesis is the final test of any theoretical molecular engineering design.

## Competing interests

The author declares that he has no competing interests.
